# Novel COL4A1‐VEGFD gene fusion in myofibroma

**DOI:** 10.1111/jcmm.16502

**Published:** 2021-04-08

**Authors:** Guillaume Dachy, Sylvie Fraitag, Boutaina Boulouadnine, Sabine Cordi, Jean‐Baptiste Demoulin

**Affiliations:** ^1^ de Duve Institute Université Catholique de Louvain Brussels Belgium; ^2^ Department of Pathology Hôpital Necker‐Enfants Malades Assistance Publique‐Hôpitaux de Paris Paris France

**Keywords:** arresten, collagen type IV, FURIN, infantile myofibromatosis, myofibroma, PDGFRB, perivascular myoid tumour, serum response factor, VEGF

## Abstract

Myofibroma is a benign pericytic tumour affecting young children. The presence of multicentric myofibromas defines infantile myofibromatosis (IMF), which is a life‐threatening condition when associated with visceral involvement. The disease pathophysiology remains poorly characterized. In this study, we performed deep RNA sequencing on eight myofibroma samples, including two from patients with IMF. We identified five different in‐frame gene fusions in six patients, including three previously described fusion transcripts*, SRF‐CITED1, SRF‐ICA1L* and *MTCH2‐FNBP4*, and a fusion of unknown significance, *FN1‐TIMP1*. We found a novel *COL4A1‐VEGFD* gene fusion in two cases, one of which also carried a *PDGFRB* mutation. We observed a robust expression of VEGFD by immunofluorescence on the corresponding tumour sections. Finally, we showed that the COL4A1‐VEGFD chimeric protein was processed to mature VEGFD growth factor by proteases, such as the FURIN proprotein convertase. In conclusion, our results unravel a new recurrent gene fusion that leads to VEGFD production under the control of the *COL4A1* gene promoter in myofibroma. This fusion is highly reminiscent of the *COL1A1‐PDGFB* oncogene associated with dermatofibrosarcoma protuberans. This work has implications for the diagnosis and, possibly, the treatment of a subset of myofibromas.

## 1 INTRODUCTION

Myofibroma are benign soft‐tissue tumours mostly found in young children. These neoplasms belong to the pericytic/perivascular tumour family in the WHO classification, based on shared histologic characteristics.^1^ The clinical presentation features solitary or multiple tumours involving the skin, muscles, bones and internal organs. The multicentric form of the disease, referred to as infantile myofibromatosis (IMF), can be a life‐threatening condition when visceral nodules are present.^2^ Unlike children, adult patients develop only solitary myofibroma.^3^

The diagnosis of myofibroma, based on pathological examination, can be challenging. Recently, we and others discovered oncogenic mutations in *PDGFRB*, encoding platelet‐derived growth factor receptor beta, a receptor tyrosine kinase which is essential for pericyte development.^3^, ^4^ In a 69‐case series, we showed that oncogenic variants of *PDGFRB* were present in 68% of IMF and 24% of isolated myofibromas. *PDGFRB* mutations have also been found in myopericytoma, a closely related entity, but not in other soft‐tissue tumours.^5^ These findings opened the possibility of treating severe myofibromatosis with tyrosine kinase inhibitors that target PDGFRB. Two such drugs, imatinib and sunitinib, showed promising results in three case reports.^6^, ^7^, ^8^ The gene alterations that drive the development of *PDGFRB* wild‐type tumours remain unclear.

Although gene fusions are frequent and potent oncogenic drivers in soft‐tissue neoplasia,^9^, ^10^ little is known regarding gene fusions in myofibroma. To date, only a few cases of myofibroma have been analysed, leading to the description of translocations involving the *SRF* gene, which encodes serum response factor. *SRF* is fused to various 3’ partner genes in soft‐tissue tumours, including *RELA* in a subset of cellular variants of myofibroma and *ICA1L* in cellular myoid neoplasms.^11^, ^12^, ^13^

The goal of this study was to perform RNA sequencing of myofibroma samples to gain insight into the genetic basis of these tumours. We confirmed the presence of *SRF* fusion transcripts. More importantly, we unravelled a new recurrent fusion gene that leads to production of the growth factor VEGFD under the control of the *COL4A1* gene promoter. This result has potential implications for the diagnosis and treatment of myofibromas.

## 2 MATERIALS AND METHODS

### 2.1 Study design

This study was approved by the medical ethics review board of the University of Louvain. We obtained archived fresh frozen samples from eight patients diagnosed with sporadic myofibroma or IMF according to the WHO classification. Some patients have already been described in a previous study.^3^

### 2.2 RNA sequencing, fusion and PDGFRB variant calling

Total RNA was extracted from fresh‐frozen tumour samples or cryomold samples (P46, P113) using TriPure reagent (Roche, Switzerland). Paired‐end RNA sequencing was performed using Illumina TruSeq Stranded mRNA libraries. Cryomold samples did not pass RNA quality thresholds and were analysed using Illumina TruSeq Exome mRNA libraries (P46 and P113). This generated at least 40 million paired‐end reads of 150 nucleotides (Macrogen, South Korea) per specimen. After a quality check using FastQC,^14^ reads (fastq files) were aligned on the GRCh37 reference genome with STAR aligner (version 2.7.2b) using two‐pass mode and optimized parameters to collect chimeric junctions, as described.^15^ Aligned sequences were then analysed for fusion calling using two different methods: STARFusion version 1.8 ^16^ and FusionCatcher version 1.20.^17^ The latter generated far more fusion candidates. We retained fusions predicted by both methods for further analysis. Variant calling was performed according to GATK best practices. RNA‐seq quantification was performed using Kallisto,^18^ followed by the Bioconductor packages tximport ^19^ and DESeq2.^20^ To perform gene set enrichment analysis, we used the Bioconductor package limma ^21^ with significantly differentially expressed genes (p‐adj <0.05) from DESeq2 analysis and the GSEA software^22^ with the whole expression data set.

### 2.3 Molecular validation of gene fusions

We selected the most promising predicted fusions, based on bioinformatic criteria (mapping quality indices) and biological relevance (in‐frame fusions involving protein‐coding genes), for molecular validation. We confirmed the presence of the gene fusions by amplifying the predicted breakpoint junction by polymerase chain reaction (PCR) after reverse transcription of tumour RNA (see Table S2 for the complete list of oligonucleotides). We cloned the fusion open reading frame of *COL4A1‐VEGFD* in pcDNA3.1/V5‐His TOPO® (according to the manufacturer's protocol; ThermoFisher Scientific) after PCR amplification of tumour complementary DNA from patient P38.

### 2.4 Plasmids and site‐directed mutagenesis

The COL4A1‐VEGFDssts mutant was produced after introduction of the mutations corresponding to VEGFD:p.R85S and p.R88S by site‐directed mutagenesis, according to the QuickChange XL‐II kit protocol (Agilent). The COL4A1‐VEGFDiiss mutant was produced after introduction of the mutations corresponding to VEGFD:p.R204S and p.R205S. We verified every construct by sequencing. We obtained mPCSK3 (FURIN) in pcDNA3.1 from Addgene (#122674, Massachussetts, USA). Full‐length human *VEGFD* in pcDNA3.1+/C‐(K)‐DYK was ordered from GenScript Biotech.

### 2.5 Histological and immunofluorescence analysis

Cryomold tumour samples were cryosectioned in 5 µm‐thick sections and immediately mounted on slides. The sections were post‐fixed with 4% paraformaldehyde and stained either with haematoxylin and eosin (HE) or for immunofluorescence (IF), as described.^23^ Tissue sections were heated for 10 min in 10 mM sodium citrate pH 6.0 for antigen retrieval. Sections were permeabilized for 5 min in 0.3% Triton X‐100 PBS solution before blocking for 1 h in 0.3% milk,10% bovine serum albumin and 0.3% Triton X‐100 in PBS. Primary antibodies were monoclonal rabbit anti‐VEGFD (ab155288; Abcam) and monoclonal mouse anti‐PDGFRB.^24^ Secondary antibodies were donkey anti‐rabbit Alexa Fluor 594 (Invitrogen # A‐21207) and donkey anti‐mouse Alexa Fluor 488 (Invitrogen # A‐21202). Primary and secondary antibodies were diluted in blocking solution and incubated at 4°C overnight and 37°C for 2 hours, respectively. Hoechst (Invitrogen) was used to stain nuclei. Fluorescence was observed with a Zeiss Axiovert 200 inverted fluorescence microscope (Zeiss). HE slides were scanned using an Oyster imaging system (3DHistech).

### 2.6 Cell culture and Western blot

COS‐1 and HEK‐293T cells were cultured in Dulbecco's Modified Eagle's Medium (DMEM, Gibco, Thermo Fisher Scientific) supplemented with 10% foetal bovine serum (FBS). Cells were seeded in 6‐well plates (400,000 cells/ well) in X‐Vivo10 serum‐free medium (Lonza) and transiently transfected with an empty vector pcDNA3, wild‐type or mutated COL4A1‐VEGFD, or VEGFD by using the calcium phosphate method.^25^ Four hours after transfection, cells were washed with X‐Vivo10 serum‐free medium. Supernatants were collected after 48 hours and centrifuged (10,000 ×*g*). Cells were washed and lysed in buffer (25 mM Tris, 0.15 M NaCl, 6 mM EDTA, 1.25 M glycerol, 1% Triton X‐100, pH 7.4, 1.7 µg/mL aprotinin, 1 mM Pefabloc and 1 mM sodium orthovanadate and 1% SDS). Western blot was performed as described,^24^ using anti‐VEGFD antibodies (ab155288).

## 3 RESULTS

### 3.1 Characterization of novel gene fusions in myofibroma

**FIGURE 1:**
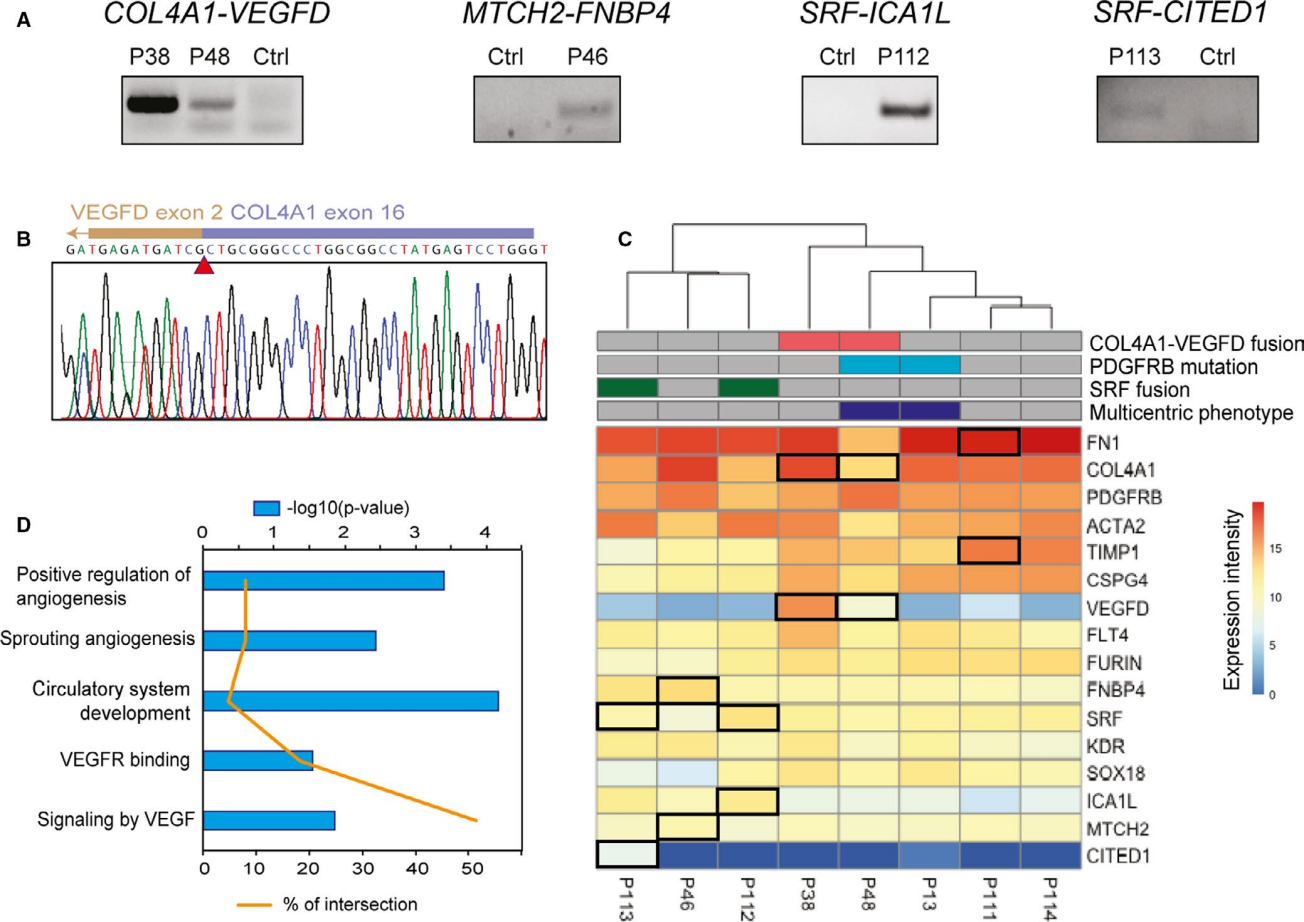
Molecular validation of novel fusion genes in myofibroma. (A) PCR amplification of breakpoint junction from tumoural cDNA. Unrelated myofibroma samples were used as controls (ctrl). (B) Sanger sequencing of the COL4A1‐VEGFD breakpoint junction amplified from cDNA. A red arrowhead indicates the intronic breakpoints. (C) Expression matrix of RNA‐seq values. The presence of a COL4A1‐VEGFD fusion, a PDGFRB mutation or a SRF fusion is indicated for each sample. Black rectangles in the expression matrix highlight the genes fused in the corresponding sample. Clustering was performed based on expression of the selected genes. See Figure S2 for further clustering analysis on larger gene sets. (D) Gene sets enriched in COL4A1‐VEGFD‐positive samples. GSEA pointed to ‘Signaling by VEGF’ (Reactome) while the limma package revealed the following gene sets: ‘Positive regulation of angiogenesis’ (GO:0045766), ‘Circulatory system development’ (GO:0072359), ‘Sprouting angiogenesis’ (GO:0002040) and ‘VEGFR binding’ (GO:0005172). P‐values are shown in blue and the percentage of the intersection between our differentially expressed genes and the gene set associated with a particular pathway in orange

We performed RNA sequencing on tumour samples from eight patients. We had previously analysed four of them by targeted sequencing of the *PDGFRB* locus.^3^ All patients were children. Two presented the multicentric form of the disease (IMF). Table 1 summarizes the patient clinical characteristics and RNA sequencing results (see also Table S1 for details). Variant calling on RNA sequencing data indicated a *PDGFRB* mutation in two patients, which had been previously reported. In addition, we detected five different in‐frame gene fusions in six patients. We validated four gene fusions by PCR amplification of the predicted breakpoint junction from tumour cDNA: *COL4A1‐VEGFD*, *SRF‐ICA1L*, *SRF‐CITED1* and *MTCH2‐FNBP4* (Figure 1A and Figure S1). The two *SRF* fusions have been recently reported in myoid neoplasms related to myofibroma,^12^, ^13^ with slightly different exon junctions (Figure S1). *MTCH2‐FNBP4* was previously described in a sample of breast cancer, without evidence of recurrence in large‐scale studies.^26^ Most of the *MTCH2* coding sequence (until exon 12) and exons 7 to 17 of *FNBP4* composed the resulting fusion gene (Figure S1). Finally, our fusion calling pipeline also identified a *FN1‐TIMP1* gene fusion of unknown significance in patient P111. The predicted *TIMP1* transcript involved in the fusion was non‐canonical, including exon 5 and part of intron 5.

**TABLE 1:** Genetic and clinical characteristics of the myofibroma samples used in the study

Patient	Fusion Name	Myofibroma type	Location	Age (year)	Gender	PDGFRB Status
P38	COL4A1‐VEGFD	Isolated	Skin, Forearm	9	F	WT
P46	MTCH2‐FNBP4	Isolated	Skin, Shoulder	0	M	WT
P48	COL4A1‐VEGFD	Multicentric	Skin	0	F	p.R561C + p.N666S
P111	FN1‐TIMP1	Isolated	Skin, Ear	0	M	WT
P112	SRF‐ICA1L	Isolated	Skin, Hallux	11	M	WT
P113	SRF‐CITED1	Isolated	Skin	15	F	WT
P114	–	Isolated	Skin	0	M	WT
P13	–	Multicentric	Skin	4	M	p.N666K + p.W566R

We focused on the *COL4A1‐VEGFD* gene fusion because it was novel, potentially oncogenic and present in two patients. RNA sequencing results indicated that the breakpoints were located in intron 17‐18 of *COL4A1* and intron 1‐2 of *VEGFD* and generated the same fusion transcript in the two patients. We amplified and cloned the full *COL4A1‐VEGFD* open reading frame from patient P38 tumour cDNA. We confirmed the predicted junction by Sanger sequencing (Figure 1B). The predicted fusion polypeptide was 643 amino‐acid long, including the 319 first residues of COL4A1 and residues 31 to 354 of VEGFD.

Quantitative analysis of RNA sequencing data showed that *VEGFD* was expressed only in myofibroma samples carrying the corresponding fusions (Figure 1C). The data also confirmed the homogeneous expression of VEGFD receptors *KDR* (VEGFR2) and *FLT4* (VEGFR3), as well as *FURIN*, a proprotein convertase that processes VEGFD.^27^, ^28^ We performed pathway enrichment analyses on the gene expression results, revealing the presence of VEGF signalling as well as angiogenesis signatures in the samples bearing the *COL4A1‐VEGFD* gene fusion, as illustrated in Figure 1D. Furthermore, the fusion was associated with a specific transcriptional profile relative to the other samples assessed (Figure S2).

### 3.2 The *COL4A1‐VEGFD* gene fusion leads to expression of mature VEGFD

**FIGURE 2:**
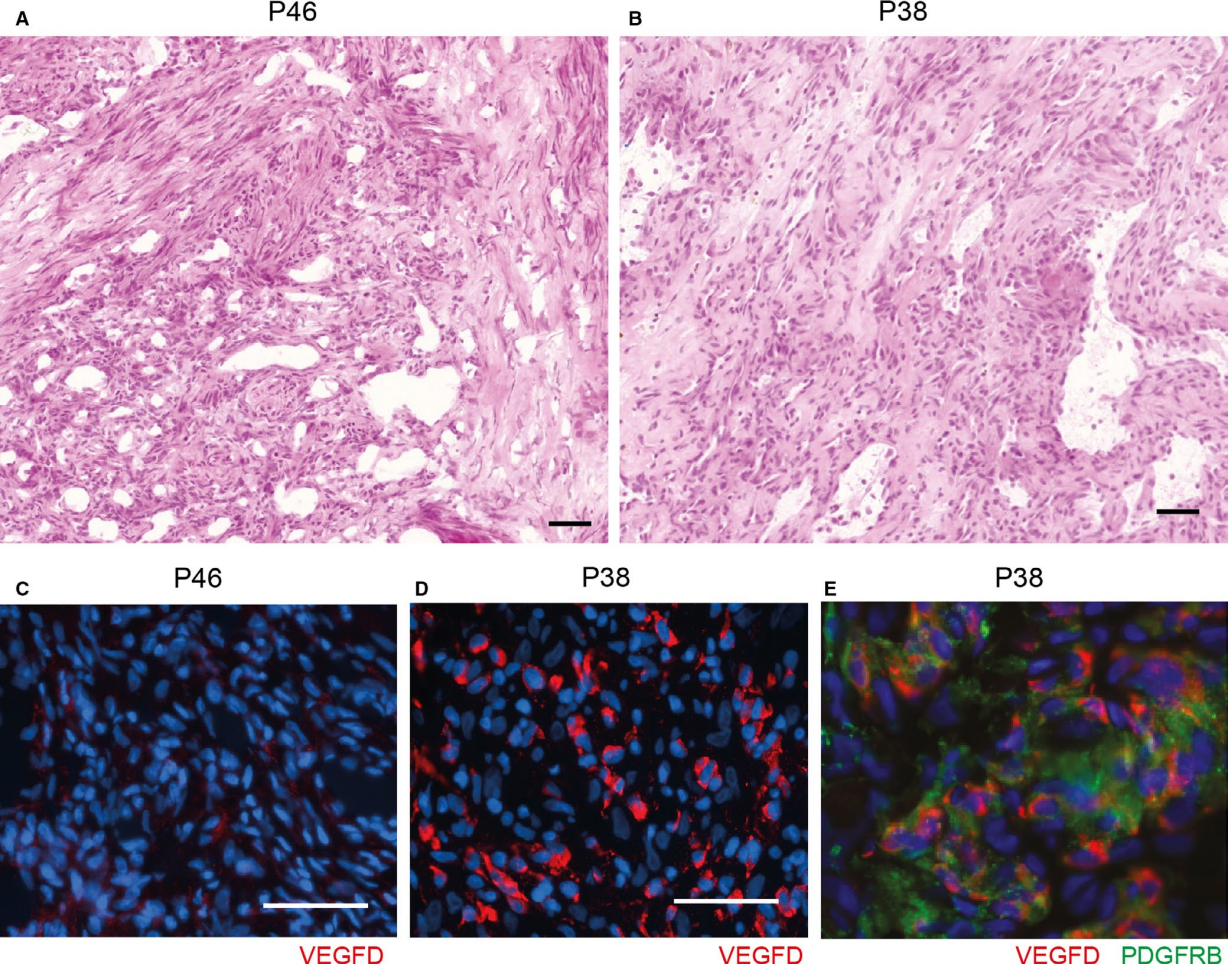
VEGFD protein expression in a COL4A1‐VEGFD‐positive tumour sample. (A, B) Microscopic features of HE stained sections of tumour samples from patients P38 and P46. (C‐E) Immunofluorescence anti‐VEGFD staining (red) of tumour tissue sections from patients P38 (COL4A1‐VEGFD‐positive) and P46 (used as a negative control). Nuclei were stained with Hoechst (blue). Scale bars correspond to 50 µm. (E) Double immunofluorescence staining targeting VEGFD (red) and PDGFRB (green)

To confirm the expression of the fusion protein, we analysed tumour sections by immunofluorescence. The haematoxylin‐eosin‐stained sections of the P38 myofibroma (Figure 2) showed classical tumoural architecture with a central haemangiopericytoma‐like vascular pattern and fascicles of myofibroblasts at the periphery.^29^ We validated the anti‐VEGFD primary antibody on transiently transfected COS‐1 cells (Figure S3). The stained tissue sections demonstrated that the expression of VEGFD was robust in the P38 tumour sample, bearing the *COL4A1‐VEGFD* gene fusion, compared to patient P46, used as negative control (Figure 2C,D). Furthermore, double staining of VEGFD and PDGFRB (a biomarker for myofibroma, myofibroblasts and pericytes) demonstrated that tumour cells expressed the two proteins (Figure 2E).

**FIGURE 3:**
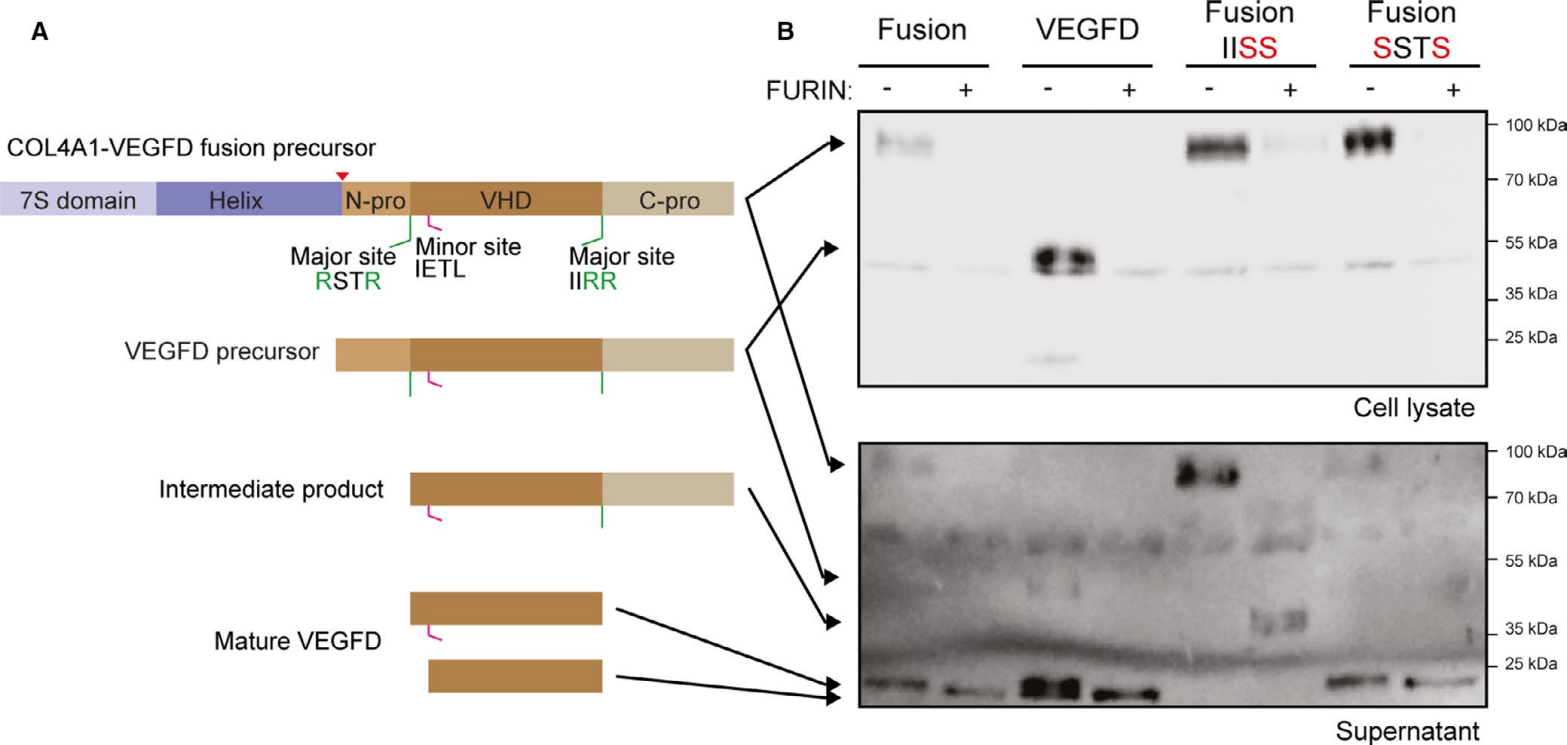
COL4A1‐VEGFD fusion protein expression and processing. (A) Schematic representation of the structures of the VEGFD and fusion propeptides, as well as the expected proteolytic products. (B) Analysis of the expression of COL4A1‐VEGFD and VEGFD by Western blot after transient transfection of HEK293T cells. Cells were cultured in the absence of serum. IISS and SSTS refer to mutations of the VEGFD major cleavage sites IIRR and RSTR, respectively. FURIN was cotransfected as indicated. The supernatant was prepared by centrifugation. One representative experiment out of three is shown

COL4A1 is composed of an N‐terminal 7S domain, a central triple‐helix‐forming (collagenous) domain and a C‐terminal non‐collagenous (NC1) domain. VEGFD is composed of a VEGF homology domain (VHD) flanked by N‐ and C‐terminal propeptides, which must be removed to produce active growth factor (Figure 3A). Seven proteases, including the proprotein convertase FURIN, have been suggested to cleave VEGFD propeptides.^30^, ^31^ Based on known cleavage sites, we predicted that the COL4A1‐VEGFD chimeric protein generates mature VEGFD, corresponding to the VHD domain (Figure 3A). To test this hypothesis, we transiently transfected *COL4A1‐VEGFD* in HEK293T cells, with or without FURIN. A plasmid encoding the VEGFD precursor protein was transfected as a positive control. We observed the full‐length precursor of COL4A1‐VEGFD (at the expected size) in lysates and supernatants of cells transfected with the fusion construct (Figure 3B). Mature VEGFD was detected in the supernatant of cells transfected with either COL4A1‐VEGFD or VEGFD, suggesting that endogenous proteases can cleave these proteins in HEK293 cells. Exogenous FURIN coexpression increased the processing efficiency (Figure 3B).

To further analyse the proteolytic processing of the fusion protein, we mutated the reported cleavage sites of VEGFD, as described^30^ (Figure 3B). As expected, the mutation of the C‐propeptide cleavage site resulted in a partially processed 40 kDa protein. Interestingly, the mutation of the N‐propeptide cleavage site alone did not affect COL4A1‐VEGFD processing to mature VEGFD. However, a COL4A1‐VEGFD chimeric protein bearing both mutated sites was not processed, even in the presence of FURIN (data not shown). The presence of an alternative cleavage site in the N‐propeptide, previously reported as a minor site,^30^ and accessible only after cleavage of the C‐propeptide could explain this observation (Figure 3A). Taken together, these results suggested that the COL4A1‐VEGFD fusion protein generates mature secreted VEGFD.

## 4 DISCUSSION

We identified a novel fusion transcript associating *COL4A1* and *VEGFD* in myofibroma. Quantitative analysis of our RNA sequencing data showed that the strong expression of *COL4A1* in myofibroma enables the expression of *VEGFD* specifically in tumours harbouring the *COL4A1‐VEGFD* fusion gene. Our results show that the COL4A1‐VEGFD chimeric protein is processed into mature VEGFD. The overexpression of a growth factor secondary to a gene fusion event is highly reminiscent of the *COL1A1‐PDGFB* translocation in dermatofibrosarcoma protuberans.^32^ The COL1A1‐PDGFB chimeric protein generates large amounts of mature PDGF‐BB, which stimulates fibroblast proliferation. The PDGF receptor inhibitor imatinib showed efficacy in COL1A1‐PDGFB‐positive dermatofibrosarcoma protuberans as adjuvant therapy, confirming the importance of the PDGF‐BB autocrine loop in tumour growth.^33^

To our knowledge, this is the first demonstration that *VEGFD* is a proto‐oncogene subject to somatic gene alteration. Nevertheless, VEGFD has previously been shown to play a role in solid tumour growth, intra‐tumoural angiogenesis, lymphangiogenesis and metastatic spread.^34^, ^35^ The analyses of the transcriptomic profiles of our samples evidenced a robust enrichment in multiple angiogenic pathways, supporting the production of bioactive VEGFD by these tumours. The fusion may also lead to reduced expression of the arresten protein, which is produced by proteolytic cleavage of the NC1 domain of COL4A1. Arresten has been described as an inhibitor of angiogenesis and a tumour suppressor, regulated by p53.^36^

The importance of the COL4A1‐VEGFD fusion should be further studied in a larger myofibroma cohort. This could lead to the validation of VEGFD as a therapeutic target for selected cases of myofibromatosis. This is of particular interest since drugs that neutralize VEGFD itself or its receptors are already available. The VEGF receptor inhibitor sunitinib has demonstrated its efficiency and safety profile in the treatment of a *PDGFRB* mutated myofibromatosis case.^7^ Other molecules developed to target VEGF receptors include pazopanib and axitinib. In addition, specific VEGFD inhibitors are currently in development and early clinical trials, including OPT‐302, a soluble form of VEGFR3 trapping VEGFD and VEGFC, and the anti‐VEGFR3 antibody IMC‐3C5.^37^, ^38^

Given the strong tumour expression of PDGFRB and histologic features reminiscent of pericytic differentiation, pericytes are the proposed cells of origin of myofibroma. It is not clear whether these cells express functional VEGF receptors.^39^, ^40^ Recent studies identified perivascular Gli1^+^ cells, which are adventitial mesenchymal stem‐like cells located in the pericyte niche in the microvasculature. These Gli1^+^ cells express typical mesenchymal stem cells (MSC) markers including PDGFRB^41^ and have the demonstrated capacity to differentiate into myofibroblasts.^42^ Remarkably, Gli1^+^ MSC‐like cells may be proangiogenic under particular conditions and able to respond to VEGF stimulation using a non‐canonical VEGF signalling pathway dependent on PDGFRB.^43^ We speculate that these mesenchymal stem‐like cells may represent the cell of origin of COL4A1‐VEGFD‐positive myofibroma, with their expression of a VEGF receptor allowing for growth stimulation by an autocrine loop.

Taking into account our previous *PDGFRB* sequencing results,^3^ we detected a putative oncogenic event in 7 of 8 myofibroma samples. The COL4A1‐VEGFD fusion was not mutually exclusive with oncogenic variants of *PDGFRB,* as shown by the co‐occurrence of *COL4A1‐VEGFD* and *PDGFRB* p.R561C/p.N666S in patient P48. In addition to *COL4A1‐VEGFD*, we detected four other gene fusions in myofibroma samples, three of which were verified by PCR amplification of the breakpoint regions in the corresponding transcripts. These results suggest that such genomic events are frequent in myofibroma, as in other soft‐tissue neoplasms.^9^ *SRF‐CITED1* and *SRF‐ICA1L* have been reported before in pericytic tumours.^12^, ^13^ These fusions were mutually exclusive with alterations in *PDGFRB* and *VEGFD*. Interestingly, SRF is a transcription factor that is activated by the mitogen‐activated protein kinase pathway, downstream of PDGFRB and VEGFD signalling.

In conclusion, we identified a novel *COL4A1‐VEGFD* fusion transcript as a recurrent genetic event. The *COL4A1‐VEGFD* fusion leads to production of mature VEGFD after proteolytic processing, which may act as an autocrine growth factor for tumour cells. These findings shed light on a novel pathogenic mechanism of myofibroma development, suggesting opportunities for targeted therapies.

## CONFLICT OF INTEREST

The authors have no potential conflict of interest to disclose.

## AUTHOR CONTRIBUTION

**Guillaume Dachy:** Conceptualization (equal); Data curation (lead); Formal analysis (lead); Funding acquisition (supporting); Investigation (lead); Methodology (equal); Project administration (equal); Writing‐original draft (equal). **Sylvie Fraitag:** Investigation (supporting); Methodology (supporting); Resources (lead); Writing‐review & editing (equal). **Boutaina Boulouadnine:** Formal analysis (supporting); Investigation (equal); Methodology (supporting); Writing‐review & editing (equal). **Sabine Cordi:** Formal analysis (supporting); Investigation (supporting); Methodology (equal); Writing‐review & editing (equal). **Jean‐Baptiste B Demoulin:** Conceptualization (equal); Data curation (supporting); Formal analysis (supporting); Funding acquisition (lead); Investigation (supporting); Methodology (supporting); Project administration (equal); Supervision (lead); Writing‐original draft (equal).

## Supporting information

Supplementary Material

## Data Availability

The data that support the findings of this study are available from the corresponding author upon reasonable request.
