# Supplementary material for: Novel COL4A1‐VEGFD gene fusion in myofibroma
Source: J Cell Mol Med. 2021 Apr 8;25(9):4387–94. doi: 10.1111/jcmm.16502 (PMC8093964; doi:10.1111/jcmm.16502)
Supplement: Supplementary file 1 — Supplementary Material [file JCMM-25-4387-s001.pdf]

Patient	Fusion Name	Sequencing coverage (Mega reads)	Junction Read Count	Spanning read pair Count	Left Breakpoint	Right Breakpoint	Involved exons	Fusion protein length (residues)
P13	-	132	-	-	-	-	-	-
P38	COL4A1-VEGFD	159	3072	552	chr13:110857700:-	chrX:15381441:-	1 to 17 (/52) - 2 to 7	643
P48	COL4A1-VEGFD	108	17	1	chr13:110857700:-	chrX:15381441:-	1 to 17 (/52) - 2 to 7	643
P46	MTCH2-FNBP4	83	27	9	chr11:47644253:-	chr11:47767946:-	1 to 12 (/13) - 7 to 17	990
P111	FN1-TIMP1	136	2	41	chr2:216259251:-	chrX:47444942:+	1 to 24 (/46) - 5 to 6	1423
P112	SRF-ICA1L	116	211	21	chr6:43146223:+	chr2:203653810:-	1 to 5 (/7) - 11 to 13	605
P113	SRF-CITED1	83	18	0	chr6:43144405:+	chrX:71522784:-	1 to 4 (/7) - 2 to 3	602
P114	-	136	-	-	-	-	-	-

Table S1. Fusion transcript data from RNA sequencing

Supplementary Table S2. List of the oligonucleotides used in the study.

<u>Aim</u>	<u>Orientation</u>	<u>Sequence</u>
COL4A1-VEGFD PCR cloning	Forward	ATGCGTCGACACCATGGGGCCCCGGCTCA
	Reverse	GCATGCGGCCGCTCAAGGATTCTTTCGGCTGTGGG
COL4A1-VEGFD junction breakpoint PCR amplification	Forward	CGGAAAACCAGGACCCAGA
	Reverse	GAAGCAGCCCTGATCTGC
COL4A1-VEGFDiiss mutagenesis	Forward	CATCCATACTCAATTATCAGCAGCTCCATCCAGATCCCTGAAG
	Reverse	CTTCAGGGATCTGGATGGAGCTGCTGATAATTGAGTATGGATG
COL4A1-VEGFDssts mutagenesis	Forward	CGCTCAGCATCCCATAGCTCCACTAGCTTTGCGGCAACTTTC
	Reverse	GAAAGTTGCCGCAAAGCTAGTGGAGCTATGGGATGCTGAGCG
MTCH2-FNBP4 junction breakpoint PCR amplification	Forward	TGCCCTCCTTACTCCCCAATATATACG
	Reverse	CAGCGATGCTGCCACCCCTT
SRF-ICA1L junction breakpoint PCR amplification	Forward	GGCAGCCTCACCGTGCTGAAT
	Reverse	GAGAATTCCTTCTCAAAATCTTCTCCTTCCA
SRF-CITED1 junction breakpoint PCR amplification	Forward	TCCCGTGACAGCAGCACAGACCT
	Reverse	AGGCCTCGACGTTGTTGGCATT

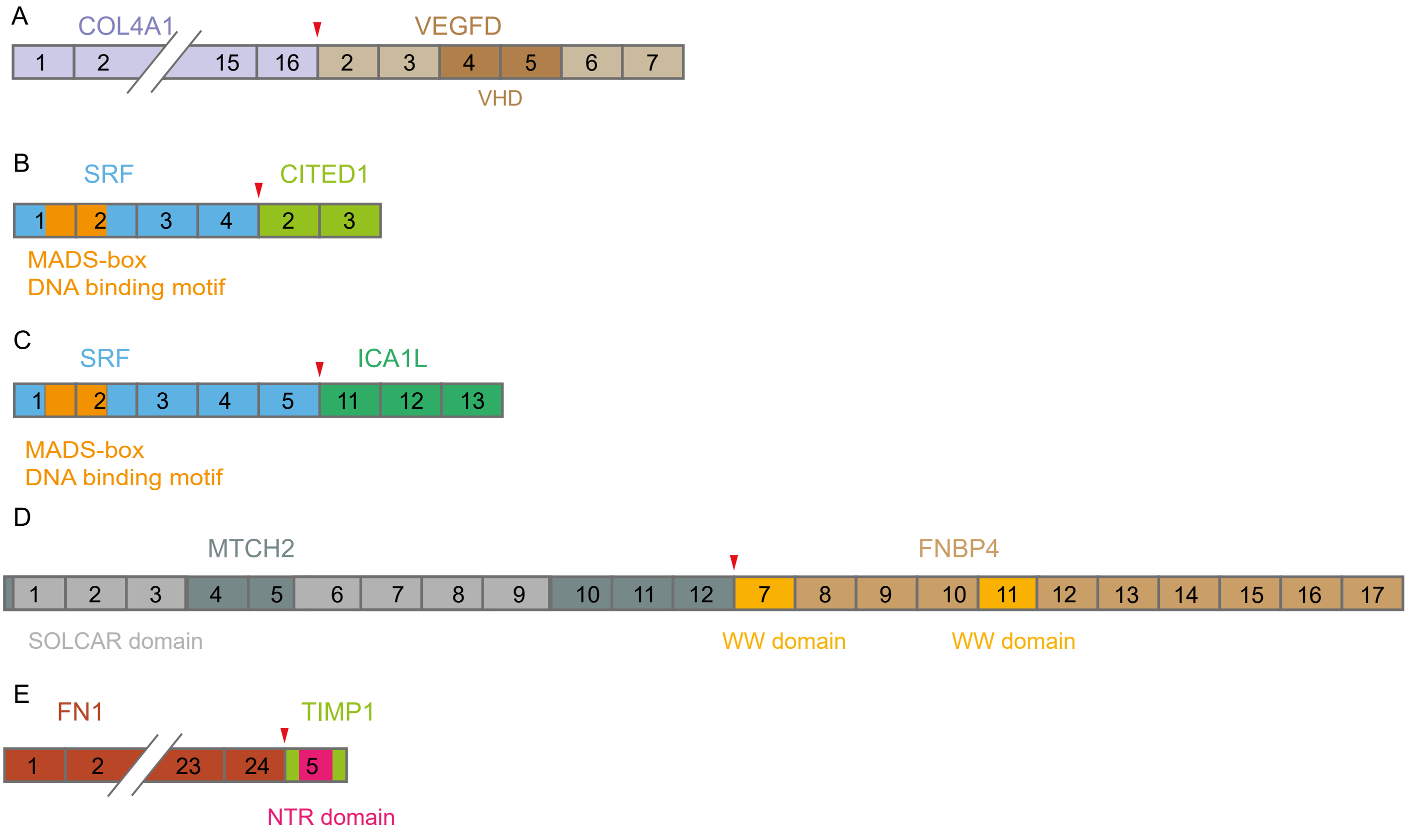

Supplementary Figure S1.

Structure of the identified fusion proteins: COL4A1-VEGFD (A), SRF-CITED1 (B), SRF-ICA1L (C), MTCH2-FNBP4 (D), FN1-TIMP1 (E). Exons encoding the fusions are indicated. Main functional domains are depicted in color. Red arrowheads indicate breakpoints.

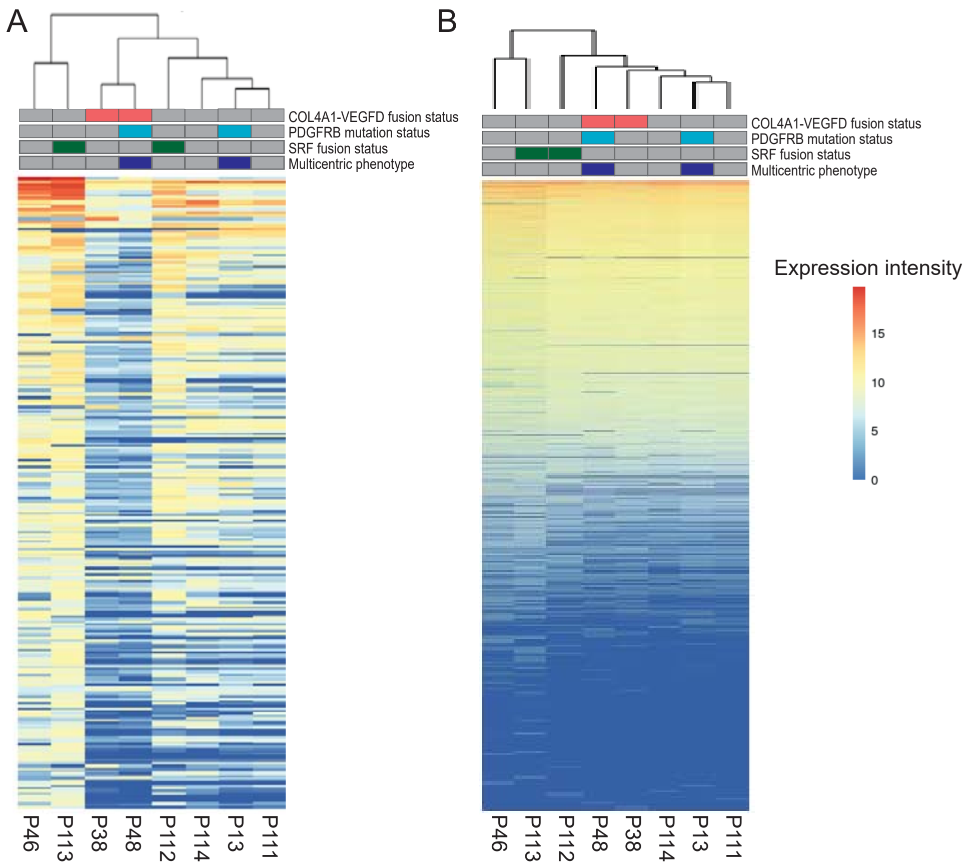

Supplementary Figure S2.

Samples harboring *COL4A1-VEGFD* fusion cluster together.
(A) Heatmap illustrating unsupervised clustering based on differentially expressed genes (p-adj< 0.05) between COL4A1-VEGFD positive and negative samples.
(B) Unsupervised clustering of samples based on all expressed genes.

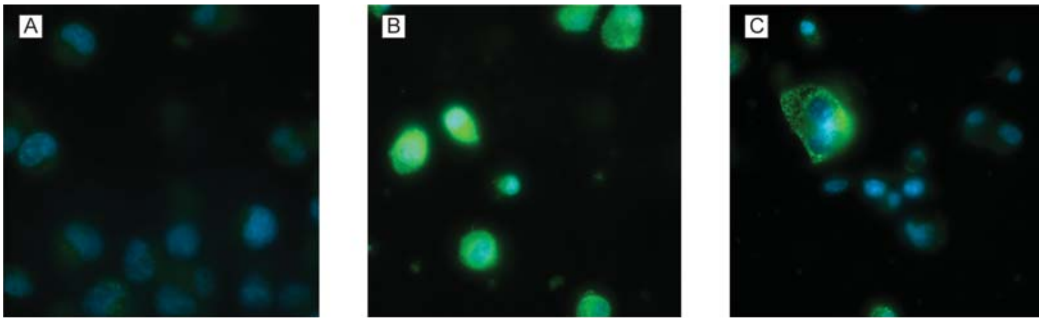

Supplementary Figure S3.

VEGFD immunofluorescence. COS-1 cells were transiently transfected with empty vector (panel A), COL4A1-VEGFD (panel B) or VEGFD (panel C). VEGFD was stained by immunofluorescence (green), as described in materials and methods. Nuclei were stained with Hoechst (blue).
